# Feasibility and Preliminary Effects of the Renkli Adımlar Game- and Drama-Based Program for Preventing Peer Bullying and Promoting Empathy: A Pilot School-Level Cluster Randomized Controlled Trial

**DOI:** 10.3390/healthcare14193182

**Published:** 2026-09-25

**Authors:** Ayşe Sezer Balcı, Sultan Günal, Rukiye Sarıca, Asiye Kaya

**Affiliations:** 1 Department of Public Health Nursing, Faculty of Health Sciences, Burdur Mehmet Akif Ersoy University, Burdur 15030, Türkiye; 2 Department of Nursing, Faculty of Health Sciences, Burdur Mehmet Akif Ersoy University, Burdur 15030, Türkiye

**Keywords:** bullying, empathy, child, schools, game-based learning, randomized controlled trial

## Abstract

**Highlights:**

**What are the main findings?**

- The Renkli Adımlar program was associated with a reduction in peer bullying victimization in the intervention school, although the single-school-per-condition design limits causal interpretation.
- The program was feasible to deliver within the participating school, with high study retention and completion of all planned intervention sessions.

**What are the implications of the main findings?**

- School-based game- and drama-based interventions may be a promising approach for addressing peer bullying victimization in primary school settings, although intervention-specific effects on empathy remain uncertain.
- The Renkli Adımlar program appears feasible to implement within a primary school context; however, broader implementation and effectiveness should be evaluated in studies including multiple schools per condition, active comparison conditions, cluster-adjusted analyses, and longer follow-up periods.

**Abstract:**

**Aim:** This pilot school-level cluster-randomized controlled trial evaluated the feasibility and preliminary effects of the Renkli Adımlar (Colorful Steps) game- and drama-based intervention on bullying and victimization, with empathy assessed as a secondary outcome, among primary school students. **Methods:** A pretest–posttest pilot trial was conducted with 79 third- and fourth-grade students allocated to an intervention group (*n* = 40) or a control group (*n* = 39). The eight-week program included six 40-min sessions using educational games, creative drama, role-playing, storytelling, puppet activities, and collaborative learning. Outcomes were assessed using the Index of Empathy for Children and Adolescents and the Peer Bullying Identification Scale–Child Form. Intervention effects were primarily examined using mixed repeated-measures ANOVA. **Results:** The mixed ANOVA showed group × time interactions for bullying (F(1, 77) = 3.959, *p* = 0.05, ηp^2^ = 0.049) and victimization (F(1, 77) = 3.870, *p* = 0.04, ηp^2^ = 0.048), with greater reductions in the intervention group than in the control group. No significant group × time interaction was observed for empathy (F(1, 77) = 0.882, *p* = 0.35, ηp^2^ = 0.011). Exploratory within-group Wilcoxon signed-rank analyses showed significant reductions in bullying and victimization and a significant increase in empathy in the intervention group, whereas no significant within-group changes were observed in the control group. **Conclusions:** The findings provide preliminary evidence that Renkli Adımlar may reduce bullying and victimization and is feasible to implement in primary schools. However, the non-significant empathy interaction does not support an intervention-specific effect on empathy. Given the inclusion of only two school clusters, findings should be considered exploratory. Larger multicenter trials with multiple schools per condition and cluster-adjusted analyses are needed. Trial registration: ClinicalTrials.gov (NCT07696455), registered retrospectively after completion of data collection.

## 1. Introduction

Peer bullying is one of the most prevalent forms of school violence and represents a major public health concern affecting children’s psychological, social, and educational development worldwide [1,2]. Bullying is characterized by unwanted aggressive behavior involving an observed or perceived imbalance of power and repeated occurrence, or a high likelihood of repetition, and may result in physical, psychological, social, or educational harm [3]. Exposure to peer bullying has been consistently associated with adverse psychosocial and mental health outcomes, including depression, anxiety, suicidal ideation and behaviors, and other psychological difficulties [4,5,6,7]. Longitudinal evidence further suggests that childhood bullying victimization may be associated with poorer mental health outcomes later in life, including depression, anxiety, and other psychiatric disorders [8,9].

Globally, approximately one in three students reports having experienced bullying at school, although prevalence varies across countries and educational contexts [2]. Recent national evidence indicates that peer bullying has become an increasingly important concern in Türkiye. According to the TIMSS 2023 Türkiye Report, the proportion of fourth-grade students reporting weekly exposure to bullying increased substantially between 2019 and 2023, whereas the proportion reporting that they had almost never experienced bullying declined considerably. These findings underscore the need for developmentally appropriate, school-based interventions to strengthen empathy, communication skills, and positive peer relationships during the primary school years [10].

Primary school is an important period for preventing bullying because children’s social, emotional, and behavioral competencies are still developing and may be responsive to environmental influences and educational interventions [11]. Accordingly, primary school may provide an important opportunity to promote positive interpersonal development and address bullying before maladaptive peer interaction patterns become more established [12]. Recent evidence strongly supports the effectiveness of universal school-based bullying-prevention programs implemented during primary education [13,14]. Among universal school-based interventions, interactive educational approaches have received increasing attention because they enable children to actively practice social–emotional competencies rather than merely acquire factual knowledge.

Bullying and victimization represent related but distinct aspects of peer bullying and should be considered as separate outcomes. Bullying refers to engaging in bullying behaviors toward peers, whereas victimization refers to experiencing such behaviors from peers. Although these roles may overlap and can influence each other over time, longitudinal evidence indicates that the relationship between perpetration and victimization is bidirectional rather than interchangeable [15,16]. Consequently, school-based interventions may not produce equivalent changes in bullying perpetration and victimization, making it important to evaluate these outcomes separately [17].

Educational interventions that combine games, creative drama, puppet role-playing, storytelling, and collaborative learning provide developmentally appropriate opportunities for primary school children to acquire social–emotional competencies through active participation. Rather than relying solely on didactic instruction, these experiential approaches enable children to practice empathy, perspective-taking, communication, and prosocial problem-solving in realistic social contexts, thereby strengthening protective skills associated with bullying prevention [12,13,18,19].

The Renkli Adımlar program was designed to influence bullying-related outcomes through several interconnected social and emotional processes. These components are consistent with the Social and Emotional Learning (SEL) framework, particularly the five core competencies identified by the Collaborative for Academic, Social, and Emotional Learning (CASEL): self-awareness, self-management, social awareness, relationship skills, and responsible decision-making [20]. Within the Renkli Adımlar program, emotional recognition and expression activities were related to self-awareness and self-management; perspective-taking, storytelling, and puppet role-playing were used to support social awareness and empathy; cooperative games and group activities provided opportunities to practice communication, cooperation, and constructive conflict resolution, consistent with relationship skills (Figure S1); and the Stop–Think–Act strategy encouraged children to pause, consider alternative responses, and reflect on the potential consequences of their actions, consistent with self-management and responsible decision-making. Perspective-taking and emotional awareness may contribute to empathy and more prosocial responses, whereas communication, relationship, and conflict resolution skills may help reduce aggressive responses associated with bullying perpetration and support more constructive responses to challenging peer interactions [21,22]. Accordingly, the intervention was expected to influence empathy, bullying perpetration, and victimization through related but potentially distinct social and emotional processes.

The theoretical rationale also suggests that bullying and victimization may respond differently to the intervention. Reductions in bullying perpetration may be more closely related to empathy and emotional self-regulation, as lower empathy has been associated with greater involvement in bullying perpetration, while emotional regulation is closely linked to broader social–emotional functioning in childhood and adolescence [21,23]. In contrast, reducing victimization may additionally involve recognizing bullying situations, communicating experiences, seeking help, and accessing support from peers or adults. Social support and willingness to seek help have been identified as relevant factors in the context of bullying victimization, while assertiveness-focused interventions have shown potential for reducing children’s experiences of victimization [18,24]. Therefore, although the intervention may influence both outcomes through overlapping social–emotional processes, the pathways through which bullying perpetration and victimization change may be partly distinct.

Recent studies conducted in Türkiye have expanded the evidence base on the prevalence, risk factors, and psychosocial correlates of peer bullying. However, intervention research remains relatively limited compared with descriptive and observational studies. Existing Turkish studies have largely employed descriptive, correlational, qualitative, or quasi-experimental designs, whereas randomized controlled evaluations of school-based bullying-prevention interventions remain limited. In particular, evidence regarding culturally tailored game-and drama-based intervention programs for primary school students remains limited [25,26,27,28].

Therefore, this pilot school-level cluster-randomized controlled trial aimed to evaluate the preliminary effectiveness and feasibility of the Renkli Adımlar game- and drama-based intervention in improving empathy and reducing bullying perpetration and victimization among primary school children. Based on the theoretical framework and previous evidence, we hypothesized that, compared with the control group, children in the Renkli Adımlar intervention group would show greater reductions in bullying and victimization from pre-test to post-test, with a greater increase in empathy as a secondary outcome.

### Research Hypotheses

**H1** **(co-primary outcome).** *Children who participate in the Renkli Adımlar intervention will show a greater reduction in peer bullying scores over time than children in the control group.*

**H2** **(co-primary outcome).** *Children who participate in the Renkli Adımlar intervention will show a greater reduction in peer victimization scores over time than children in the control group.*

**H3** **(secondary outcome).** *Children who participate in the Renkli Adımlar intervention will show a greater increase in empathy scores over time than children in the control group.*

## 2. Methods

### 2.1. Study Design

This study was designed as a pilot school-level cluster-randomized controlled trial with a pretest–posttest parallel-group design to evaluate the preliminary effectiveness and feasibility of the Renkli Adımlar (Colorful Steps) game- and drama-based intervention in promoting empathy and reducing bullying perpetration and victimization among primary school children. Because the intervention was delivered within intact classroom environments, randomization was performed at the school (cluster) level to minimize contamination between participants. Given that only two school clusters were included, the trial was primarily designed to evaluate feasibility and generate preliminary estimates of intervention effects rather than provide definitive evidence of effectiveness. The study was reported in accordance with the CONSORT 2010 Statement, the CONSORT extension for cluster-randomized trials, and the CONSORT extension for randomized pilot and feasibility trials.

### 2.2. Recruitment

The target population consisted of third- and fourth-grade students enrolled in public primary schools. Students were eligible if they were enrolled in the selected schools, attended regular classes, and provided written parental consent together with verbal child assent. Of the 84 students assessed for eligibility, 5 were excluded because parental consent was not obtained, leaving 79 students who participated in the trial. All 79 participating students completed follow-up and were included in the primary analysis, corresponding to a participation rate of 94.0% and a retention rate of 100% (Figure 1).

### 2.3. Study Setting and Participants

Following school-level randomization, class lists were obtained from each participating school. Two third-grade classes and two fourth-grade classes were selected from each school using a computerized random selection procedure. Students in the selected classes were assessed for eligibility according to the predefined criteria. Students who met the eligibility criteria and provided written parental consent, along with verbal child assent, were included in the study. The intervention was delivered to students in the school allocated to the intervention condition, whereas students in the school allocated to the control condition continued their usual school activities throughout the study period.

### 2.4. Sample Size and Randomization

#### 2.4.1. Sample Size

An a priori sample size calculation was performed using G*Power version 3.1.9.7 (Heinrich Heine University Düsseldorf, Germany), assuming an individually randomized design with a medium effect size (Cohen’s *d* = 0.65), a two-sided significance level of 0.05, and 80% statistical power (1 − β = 0.80). Because the present study used school-level cluster-randomization and included only two randomized school clusters, the calculation did not incorporate an intracluster correlation coefficient (ICC) or a cluster design effect. Therefore, the resulting sample size was considered a pragmatic estimate for planning this pilot study rather than a definitive sample size calculation for a confirmatory cluster-randomized trial.

#### 2.4.2. Randomization

Following school recruitment, the two participating schools were randomly allocated in a 1:1 ratio to the intervention or control group using a computer-generated random sequence created in Microsoft Excel by the first author. Randomization was performed at the school (cluster) level before participant enrollment to minimize potential contamination between study groups. Because allocation was performed at the school (cluster) level before participant enrollment, allocation concealment at the individual participant level was not applicable. Following school-level allocation, class lists were obtained from each participating school. Two third-grade classes and two fourth-grade classes were selected from each school using a computerized random selection procedure. Students in the selected classes were then assessed for eligibility according to the predefined inclusion criteria, and those who provided written parental consent and verbal child assent were enrolled in the study.

#### 2.4.3. Blinding

Owing to the nature of the educational intervention, blinding of participants, classroom teachers, and the researcher responsible for delivering the intervention was not feasible. To minimize the risk of assessment and analytical bias, standardized outcome measures were administered under identical conditions in both study groups at baseline and immediately after the intervention. Participants completed the questionnaires using unique study identification numbers, and all personally identifiable information was removed before data entry. Data was entered into a password-protected electronic database using anonymous participant identifiers. Statistical analyses were conducted using datasets containing coded group assignments rather than descriptive group labels. Group allocation codes were revealed only after completion of the primary statistical analyses. Because randomization was performed at the school (cluster) level, allocating different schools to the intervention and control conditions also minimized contamination between participants by reducing opportunities for information exchange regarding intervention activities. Despite these precautions, the absence of assessor blinding should be considered when interpreting the findings because complete blinding was not feasible in an educational intervention delivered at the classroom level.

### 2.5. Intervention Fidelity

All intervention sessions were delivered by the second, third, and fourth authors, who followed the standardized intervention manual and completed the implementation checklist after each session. The first author monitored the session content and implementation to ensure adherence to the intervention protocol. Before implementation, each session was pilot-tested and reviewed by experts in child health nursing, educational sciences, and school counseling. Following every session, a structured implementation checklist was completed to verify adherence to the planned learning objectives, teaching methods, and educational materials. Attendance was recorded for each session, and any deviations from the intervention protocol were documented. 

### 2.6. Data Collection Tools

Data were collected using the Peer Bullying Identification Scale–Child Form (PBIS-CF), the Index of Empathy for Children and Adolescents (IECA), and a Personal Information Form.

#### 2.6.1. Peer Bullying Identification Scale–Child Form (PBIS-CF):

Peer bullying perpetration and victimization were assessed using the Peer Bullying Identification Scale–Child Form (PBIS-CF) developed by Piskin and Ayas (2011) [29]. The instrument consists of 37 items assessing both bullying and victimization across five dimensions: physical bullying, verbal bullying, social exclusion, rumor spreading, and damage to belongings. Each behavior is rated separately for perpetration and victimization using a 5-point Likert-type frequency scale, ranging from 0 (never) to 4 (almost every day). Total scores range from 37 to 148 for both the bullying and victimization subscales, with higher scores indicating greater involvement in bullying perpetration or victimization. In the original validation study, the internal consistency coefficients (Cronbach’s α) were 0.87 for the bullying subscale and 0.90 for the victimization subscale, indicating excellent reliability. In the present study, the internal consistency of the PBIS-CF subscales was assessed using both Cronbach’s α and McDonald’s ω. Reliability coefficients were calculated separately at baseline and post-test. Cronbach’s α coefficients for the bullying subscale were 0.89 at baseline and 0.84 at post-test, Cronbach’s α coefficients for the victimization subscale were 0.92 at baseline and 0.87 at post-test. McDonald’s ω coefficients were (0.88) and (0.83) for bullying and (0.91) and (0.86) for victimization at baseline and post-test, respectively.

#### 2.6.2. Index of Empathy for Children and Adolescents (IECA)

Empathy was measured using the Index of Empathy for Children and Adolescents (IECA), as adapted into Turkish by Gürtunca (2013) [30]. The scale evaluates children’s ability to understand and share others’ emotions. The original instrument consists of 22 dichotomous (Yes/No) items assessing affective empathy. The Turkish adaptation conducted by Gürtunca (2013) removed one item, resulting in a 21-item version [30]. Higher total scores indicate greater empathy. The Turkish version demonstrated satisfactory psychometric properties, with a KR-20 coefficient of 0.70 and a test–retest reliability coefficient of 0.76, supporting its validity and reliability for use with Turkish children and adolescents. In the present study, the internal consistency of the IECA was assessed using the Kuder–Richardson Formula 20 (KR-20), and the corresponding 95% confidence interval was calculated. The reliability estimate was 0.71 (95% CI: 0.51–0.73).

#### 2.6.3. Personal Information Form

A researcher-developed Personal Information Form was used to obtain participants’ demographic characteristics, including age, sex, grade level, and number of siblings.

### 2.7. Intervention: The Renkli Adımlar (Colorful Steps) Program

The Renkli Adımlar (Colorful Steps) program is a researcher-developed, manualized, school-based intervention designed to reduce peer bullying and promote empathy among primary school children through interactive social–emotional learning activities. The intervention was theoretically informed by the Social and Emotional Learning (SEL) framework and contemporary evidence on school-based bullying prevention. Educational content was specifically designed to strengthen empathy, emotional awareness, perspective-taking, communication, cooperation, responsible decision-making, and constructive conflict resolution. The program was delivered over eight consecutive weeks, with one 40-min educational session per week during Weeks 2–7. Week 1 was dedicated to orientation and baseline assessment, whereas Week 8 included the post-test assessment and program closure (Table 1). Week 1 was devoted to participant orientation and baseline assessment, Weeks 2–7 comprised the six structured educational modules, and Week 8 included the post-intervention assessment, program review, and closure activities. The six educational modules incorporated educational games, creative drama, role-playing, storytelling, puppet-assisted activities, cooperative learning, and structured classroom discussions, as described in Table 1.

### 2.8. Intervention Fidelity and Attendance

All six planned intervention sessions were delivered as scheduled. Attendance was monitored throughout the intervention period, and participants included in the intervention analysis attended the intervention sessions regularly. No major deviations from the planned intervention protocol were documented. No intervention-related adverse events were reported.

### 2.9. Feasibility and Acceptability Assessment

Feasibility and acceptability were assessed using predefined process indicators covering recruitment, retention, attendance, intervention delivery, implementation fidelity, protocol deviations, adverse events, and participant and teacher feedback. Recruitment feasibility was assessed by the proportion of eligible students who consented to participate. Retention was assessed by the proportion of enrolled participants who completed the post-intervention assessment. Attendance was assessed for each intervention session using session attendance records. Intervention dose was defined as the number of planned intervention sessions attended by each participant. Intervention delivery and adherence to the intervention protocol were assessed using a structured implementation checklist completed after each session, covering the planned learning objectives, teaching methods, and educational materials. Acceptability was assessed descriptively using written feedback from participating teachers regarding student engagement, perceived usefulness, observed behavioral changes, and suggestions for future implementation. Adverse events occurring during the intervention period were monitored and documented.

### 2.10. Data Collection Procedure

Data collection was conducted between February and March 2026 in two phases: baseline (pre-test) and post-intervention (post-test). Prior to data collection, the study’s purpose and procedures were explained to school administrators, classroom teachers, parents or legal guardians, and students. At baseline, students in both the intervention and control groups completed the Personal Information Form, the Index of Empathy for Children and Adolescents (IECA), and the Peer Bullying Identification Scale–Child Form (PBIS-CF) under standardized classroom conditions. All assessments were administered by the principal researcher using identical procedures in both groups.

Following the baseline assessment, students in the intervention group participated in the six-session *Renkli Adımlar* program. During the same period, students in the control group continued their standard curriculum provided by the Ministry of National Education and did not receive any structured bullying-prevention intervention.

Immediately after completion of the intervention, students in both groups completed the same assessment instruments under conditions identical to those used at baseline. To ensure confidentiality, each participant was assigned a unique study identification number, and all questionnaires were anonymized before data entry and statistical analysis. Data were entered into an electronic database using coded participant identifiers, and statistical analyses were performed using de-identified datasets with coded group assignments. After completion of the post-test assessment, students in the control group were offered a condensed two-session version of the *Renkli Adımlar* program to ensure equitable access to the intervention.

### 2.11. Ethical Issues

Ethical approval for this study was obtained from the Non-Interventional Clinical Research Ethics Committee of Burdur Mehmet Akif Ersoy University (Approval No.: GO 2025/2340; 10 December 2025) prior to the commencement of the study. Written informed consent was obtained from the parents or legal guardians of all participating students before enrollment. In addition, the study procedures were explained to the children in age-appropriate language, and their verbal assent was obtained prior to participation. Participation was entirely voluntary, and both parents and children were informed that they could withdraw from the study at any time without negative consequences. All intervention activities were educational, age-appropriate, and posed no foreseeable physical or psychological risks to the participating children.

### 2.12. Data Analysis

Statistical analyses were performed using IBM SPSS Statistics version 26.0 (IBM Corp., Armonk, NY, USA). Descriptive statistics were calculated for all study variables. Continuous variables are presented as means and standard deviations (SD), whereas categorical variables are summarized as frequencies and percentages. Baseline comparability between the intervention and control groups was assessed using the Mann–Whitney U test for continuous variables and the chi-square test for categorical variables, as appropriate.

The prespecified primary outcome was peer bullying, assessed using the Peer Bullying Identification Scale–Child Form (PBIS-CF), with bullying perpetration and victimization examined as separate subscales. Empathy, assessed using the Index of Empathy for Children and Adolescents (IECA), was prespecified as a secondary outcome. This outcome hierarchy was prespecified before data collection, as documented in the ethics committee research application submitted on 9 December 2025. The ClinicalTrials.gov registration (NCT07696455) was completed retrospectively after data collection was complete. The retrospective registration resulted from an administrative oversight in the study registration process.

To evaluate changes in outcomes over time, a two-way mixed analysis of variance (mixed ANOVA) was performed, with group (intervention vs. control) as the between-subjects factor and time (pre-test vs. post-test) as the within-subjects factor. The group × time interaction was used to assess differential change between the intervention and control groups for the prespecified primary outcome and its bullying and victimization subscales. Empathy was analyzed as a secondary outcome. Effect sizes were reported as partial eta squared (ηp^2^) and interpreted according to Cohen’s recommendations (0.01 = small, 0.06 = medium, and 0.14 = large). Before conducting the mixed ANOVA, assumptions of normality (Shapiro–Wilk test) and homogeneity of variances (Levene’s test) were assessed. The Shapiro–Wilk tests indicated departures from normality for all outcome measurements (*p* < 0.05). Levene’s test indicated homogeneity of variances for most assessments, except for baseline victimization (F(1,77) = 8.168, *p* = 0.005). Given the pilot nature of the study and the limited number of randomized school clusters, the mixed ANOVA was retained as the prespecified primary analysis of the group × time interaction and was interpreted as exploratory and hypothesis-generating. Because several outcomes were not normally distributed, Wilcoxon signed-rank tests were additionally conducted as supportive exploratory within-group analyses rather than as replacements for the primary group × time analysis. Although randomization was performed at the school (cluster) level, only two schools were included, with one school assigned to each study condition. Therefore, school-level clustering could not be separated from intervention assignment, and reliable estimation of the intracluster correlation coefficient (ICC) or implementation of cluster-adjusted analyses, such as multilevel modeling or generalized estimating equations, was not feasible. Accordingly, the mixed ANOVA analyses were conducted at the individual participant level and were interpreted as exploratory and hypothesis-generating.

Because several outcome variables were not normally distributed and the sample size was relatively small, Wilcoxon signed-rank tests were also performed to examine within-group pre-test-to-post-test changes. These analyses were considered supportive exploratory analyses rather than primary tests of intervention effectiveness. Effect sizes for the Wilcoxon analyses were calculated as r = Z/√N and interpreted as small (0.10), medium (0.30), or large (0.50). Given the increased risk of Type I error associated with multiple within-group comparisons, Wilcoxon results were interpreted cautiously and used only to complement the primary mixed ANOVA findings. The internal consistency of the PBIS-CF bullying perpetration and victimization subscales was assessed using both Cronbach’s alpha (α) and McDonald’s omega (ω). Reliability coefficients were calculated separately for the baseline and post-test assessments to evaluate the internal consistency of each subscale at both measurement points. All statistical tests were two-sided, and a *p*-value ≤ 0.05 was considered statistically significant.

## 3. Results

### 3.1. Participant Recruitment and Characteristics

A total of 79 primary school students participated in the study, including 40 students in the intervention group and 39 students in the control group. The baseline demographic and clinical characteristics of the participants are presented in Table 2. There were no statistically significant differences between the intervention and control groups regarding gender distribution (χ^2^ = 0.30, *p* = 0.58), maternal educational level (χ^2^ = 2.29, *p* = 0.31), paternal educational level (χ^2^ = 1.32, *p* = 0.51), or number of siblings (χ^2^ = 2.45, *p* = 0.65). Similarly, the groups did not differ significantly with respect to age (9.37 ± 0.58 vs. 9.46 ± 0.60 years, *Z* = −0.65, *p* = 0.51), empathy scores measured by the Index of Empathy for Children and Adolescents (IECA) (*Z* = −0.62, *p* = 0.52), PBIS-CF bullying scores (*Z* = −0.97, *p* = 0.37), or PBIS-CF victimization scores (*Z* = −1.76, *p* = 0.07) (Table 2).

### 3.2. Primary Analysis

For peer bullying, the group × time interaction reached the prespecified statistical significance threshold of *p* ≤ 0.05, *F*(1,77) = 3.959, *p* = 0.050, ηp^2^ = 0.049. Bullying scores decreased from 10.65 ± 14.02 to 7.58 ± 10.57 in the intervention group (mean change = −3.08), whereas they increased from 10.15 ± 16.36 to 10.74 ± 16.58 in the control group (mean change = +0.59). The between-group difference in change was −3.66 (95% CI: −7.32 to −0.01). For peer victimization, the group × time interaction was statistically significant, *F*(1,77) = 3.870, *p* = 0.040, ηp^2^ = 0.048. Victimization scores decreased from 24.88 ± 24.56 to 15.52 ± 16.53 in the intervention group (mean change = −9.35), compared with a decrease from 14.30 ± 12.34 to 13.30 ± 11.12 in the control group (mean change = −1.00). The between-group difference in change was −8.35 (95% CI: −16.80 to −0.10). For empathy, the group × time interaction was not statistically significant, *F*(1,77) = 0.882, *p* = 0.351, ηp^2^ = 0.011. Empathy scores increased from 6.78 ± 2.06 to 7.17 ± 2.71 in the intervention group (mean change = +0.39) and from 6.58 ± 2.18 to 7.02 ± 2.21 in the control group (mean change = +0.44). The between-group difference in change was −0.05 (95% CI: −0.16 to 0.06) (Table 3). Changes in empathy, victimization, and bullying scores over time in the intervention and control groups are illustrated in Figure 2.

### 3.3. Secondary Analysis: Within-Group Analyses

Within-group analyses showed that bullying scores significantly decreased in the intervention group (Z = −2.13, *p* = 0.033, *r* = 0.34), whereas no statistically significant change was observed in the control group (Z = −1.29, *p* = 0.199, *r* = 0.20). Similarly, victimization scores significantly decreased in the intervention group (Z = −2.80, *p* = 0.004, *r* = 0.44), whereas no statistically significant change was observed in the control group (Z = −0.54, *p* = 0.588, *r* = 0.09). For empathy, a significant pre-test-to-post-test increase was observed in the intervention group (Z = −2.38, *p* = 0.017, *r* = 0.38), whereas the change in the control group was not statistically significant (Z = −1.45, *p* = 0.143, *r* = 0.23). These within-group findings were considered supportive and exploratory and do not replace the primary group × time comparisons from the mixed repeated-measures ANOVA (Table 4).

### 3.4. Process Evaluation

At the completion of the intervention, feedback was obtained from two teachers through open-ended questions. The responses were descriptively organized into common categories reflecting teachers’ observations of student engagement, the appropriateness of the activities, and the implementation process. No formal qualitative analysis was conducted. The qualitative feedback obtained from the two teachers was summarized by frequency. Both teachers (2/2) reported that the intervention increased students’ awareness of bullying. Both teachers (2/2) considered the puppet-based role-play activities particularly effective in engaging students and facilitating learning. Both teachers (2/2) recommended extending the duration of the intervention to allow more time for activities and discussion.

Teachers’ feedback was summarized into three descriptive categories: (1) increased awareness of bullying, (2) the effectiveness of puppet-based role-playing activities, and (3) recommendations regarding the duration of the program. Teachers consistently reported that the intervention increased students’ awareness of bullying and helped them distinguish bullying behaviors from ordinary peer conflicts. As one teacher stated, “Students began to recognize that some behaviors they previously considered joking were actually forms of bullying.” (Teacher 1). Teachers also emphasized that students became more willing to discuss their experiences with bullying and appropriate responses during classroom activities. Among all intervention components, puppet-assisted role-playing was consistently identified as the most influential activity. According to one teacher, “The puppet activities attracted students’ attention the most and helped them understand how the victim might feel.” (Teacher 2). Teachers explained that dramatizing the roles of both the bully and the victim encouraged children to consider different perspectives, recognize the emotional consequences of bullying, and participate more actively in classroom discussions. They perceived this activity as particularly effective for fostering empathy and strengthening children’s understanding of appropriate peer interactions. Finally, teachers highlighted the game- and drama-based structure of the program as a key factor in maintaining students’ attention and encouraging active participation throughout the intervention. Although they considered the six-session program feasible for implementation in routine primary school education, they suggested that extending its duration could further strengthen empathy and promote more sustainable improvements in peer relationships and bullying prevention.

### 3.5. Intervention Attendance and Implementation Fidelity

All six planned educational intervention sessions were delivered as scheduled by the second, third, and fourth authors. The first author supervised and monitored implementation throughout the intervention period. According to the implementation checklists completed after each session, all planned intervention activities were delivered as specified in the intervention protocol, with no deviations from the planned intervention content or delivery schedule. The intervention group included 40 students, all of whom attended all six sessions (100% attendance).

## 4. Discussion

The mixed ANOVA showed a group × time interaction for bullying, with bullying scores decreasing in the intervention group. A similar decrease was observed in the supportive within-group analysis; however, given the limited number of randomized school clusters and the pilot nature of the study, this finding should be interpreted cautiously as preliminary evidence. The mixed ANOVA demonstrated a statistically significant group × time interaction for victimization, with victimization scores decreasing in the intervention group. Although a significant within-group reduction was also observed, this finding should likewise be interpreted cautiously given the limited number of randomized school clusters and the higher baseline victimization scores in the intervention group.

For empathy, the primary mixed repeated-measures ANOVA did not demonstrate a statistically significant group × time interaction, indicating that changes in empathy did not differ significantly between the intervention and control groups. Although empathy scores increased significantly within the intervention group in the supportive Wilcoxon analysis, the control group also showed an increase in mean empathy scores, and the between-group difference in change was not statistically significant. Therefore, the within-group increase should not be interpreted as evidence of an intervention-specific effect on empathy. The observed increase may reflect factors unrelated to the intervention, such as common classroom experiences, temporal changes, or other contextual influences. Accordingly, the present study does not provide evidence of an intervention-specific effect on empathy, and this outcome should be examined in adequately powered future cluster-randomized trials involving multiple schools per condition.

This pattern is broadly consistent with previous school-based social and emotional learning research, in which improvements in empathy have not necessarily translated into statistically significant intervention-specific effects relative to control conditions. For example, Xavier et al. (2023) reported an increase in empathy following a school-based compassion-focused social–emotional learning intervention, but the group × time interaction was not statistically significant, indicating that the observed change could not be clearly attributed to the intervention relative to the comparison condition [31]. More broadly, a large meta-analysis of universal school-based SEL interventions found positive effects across a range of social–emotional and behavioral outcomes, with substantial heterogeneity across intervention characteristics, implementation contexts, and outcome domains [32]. These findings suggest that intervention effects may vary across specific social–emotional competencies and that improvements in one domain should not necessarily be expected uniformly across all outcomes.

Several theoretically plausible explanations may account for the absence of a differential effect on empathy. The duration and intensity of the intervention may not have been sufficient to produce measurable changes in affective empathy within the study period. In addition, the IECA may not have captured some of the specific social–emotional skills targeted by the intervention, such as perspective-taking, emotional recognition, relationship skills, and help-seeking. These possibilities are consistent with the program’s SEL-oriented content but were not directly examined in the present study and should therefore be regarded as tentative interpretations rather than conclusions supported by the data.

Another possible explanation is that empathy was not targeted as a dedicated intervention component. Although the Renkli Adımlar program included perspective-taking, emotional recognition, storytelling, puppet role-play, cooperative learning, and discussion of interpersonal situations, these activities were embedded within a broader multicomponent bullying-prevention program rather than delivered as a focused empathy intervention. Thus, the program provided opportunities for children to engage with empathic processes, but the frequency, intensity, and explicitness of this practice may not have been sufficient to produce measurable changes in empathy over the six-week intervention period. Future versions of the program could strengthen the empathy component through systematic perspective-taking exercises, explicit emotion-recognition activities, guided reflection following role-play, and repeated discussion of the feelings and perspectives of children involved in bullying situations. The effectiveness of these strategies should be evaluated empirically in future studies.

The mixed ANOVA showed group × time interactions for bullying and victimization, with greater reductions in the intervention group than in the control group. For bullying, scores decreased in the intervention group, and a similar decrease was observed in the supportive within-group analysis. However, given the limited number of randomized school clusters, this finding should be interpreted cautiously as preliminary evidence. The mixed ANOVA also demonstrated a statistically significant group × time interaction for victimization, with victimization scores decreasing in the intervention group. Although a significant within-group reduction was observed, this finding should likewise be interpreted cautiously given the limited number of school clusters and the higher baseline victimization scores in the intervention group. This pattern is consistent with previous studies showing that school-based interventions incorporating games, role-play, cooperative learning, and creative drama can reduce aggressive behaviors by improving emotional regulation, perspective-taking, and peer relationships. For example, Avşar and Ayaz-Alkaya (2024) reported that a multi-model peer advocacy intervention improved students’ bullying-prevention skills, self-confidence, and awareness of effective anti-bullying strategies, while Şahin and Ayaz-Alkaya (2024) found that a randomized controlled motivational interviewing intervention significantly reduced bullying behaviors among adolescents [33,34]. Similarly, the recent UK multicenter cluster-randomized controlled trial of the KiVa anti-bullying program demonstrated a significant reduction in bullying victimization among primary school students, although its effects on bullying perpetration were comparatively smaller [35]. These findings are further supported by recent evidence syntheses indicating that school-based anti-bullying interventions generally produce small-to-moderate rather than large behavioral changes, reflecting the multifaceted nature of bullying, which is shaped by individual, peer, family, classroom, and school-level influences. The small-to-moderate effect sizes observed in the present study are consistent with the updated meta-analysis by Gaffney et al. (2021), which concluded that school-based bullying-prevention programs generally produce modest improvements in bullying perpetration and victimization [17]. Accordingly, the effect sizes observed in the present pilot trial are comparable to those reported in the broader international literature.

More broadly, the present findings are consistent with evidence supporting school-based social and emotional learning and bullying-prevention interventions. In a contemporary meta-analysis of 424 universal school-based SEL studies involving more than 575,000 students, Cipriano et al. (2023) reported improvements in social–emotional skills, attitudes, behaviors, peer relationships, school climate and safety, and other school-related outcomes, while also identifying substantial heterogeneity across intervention characteristics, contexts, and implementation quality [32]. This broader evidence base is relevant to the present intervention because Renkli Adımlar similarly integrates social–emotional learning with structured, participatory classroom activities. Evidence from bullying-prevention research also indicates that school-based interventions can reduce bullying and promote prosocial outcomes, although the magnitude and consistency of effects vary across programs and populations [17,36]. More recently, an umbrella review synthesizing 30 meta-analyses reported overall beneficial effects of bullying-prevention interventions while highlighting variation across intervention approaches and outcome domains [37]. Taken together, these findings broadly align with previous evidence, particularly regarding victimization, but should be interpreted as preliminary given that the present study included only two school clusters and did not use an active comparison condition. The study, therefore, provides a pilot contribution to the existing literature rather than confirmatory evidence of intervention effectiveness.

A statistically significant group × time interaction was observed for victimization, with children in the intervention group showing a reduction in victimization scores from pre-test to post-test. The intervention incorporated creative drama, educational games, puppet-assisted role-playing, storytelling, cooperative learning, and structured classroom discussions, through which children were provided opportunities to recognize bullying situations, express emotions, seek social support, and practice constructive responses to interpersonal conflicts. These intervention components are consistent with approaches used in school-based social–emotional learning and bullying-prevention programs, in which participatory activities, role-play, cooperative learning, and structured peer interaction provide opportunities to practice social responses and manage challenging interpersonal situations [17,32]. Creative drama and role-play may also facilitate perspective-taking and behavioral rehearsal by allowing children to explore interpersonal situations and practice alternative responses in a structured setting [38,39]. Puppet-assisted activities may provide developmentally appropriate opportunities to explore emotions and social interactions and have been associated with social–emotional development in young children [40,41]. Cooperative learning and structured peer interaction may similarly support opportunities to practice communication, cooperation, and prosocial responses [42]. However, the extent to which any individual component contributed to the observed reduction in victimization cannot be determined because the present study evaluated a multicomponent intervention and did not examine component-specific effects.

These intervention components can be interpreted through the Social and Emotional Learning (SEL) framework, particularly its core competencies: self-awareness, self-management, social awareness, relationship skills, and responsible decision-making [20,32]. For example, emotional recognition and expression are relevant to self-awareness and self-management; perspective-taking and recognition of others’ experiences are related to social awareness; and cooperative activities and constructive conflict resolution are consistent with relationship skills. Similarly, activities such as Stop–Think–Act encourage children to consider alternative behavioral responses and potential consequences, corresponding to self-management and responsible decision-making. These processes may provide plausible pathways through which an SEL-oriented intervention could influence responses to problematic peer interactions. However, the present study directly assessed bullying perpetration, bullying victimization, and empathy, whereas emotional regulation, responsible decision-making, relationship skills, perspective-taking, and help-seeking were not assessed as separate outcomes or mediating mechanisms. Therefore, these mechanisms should be regarded as theoretically informed interpretations rather than demonstrated explanations or mediating pathways. Future research should measure these potential mechanisms explicitly and examine whether changes in specific SEL competencies account for changes in bullying-related behaviors.

Although the baseline difference in victimization between the intervention and control groups did not reach statistical significance, the intervention group had a substantially higher mean victimization score at baseline. This imbalance is important when interpreting the subsequent reduction in victimization because the intervention group had greater scope for improvement and regression to the mean may have contributed to the observed decrease. In addition, because each study condition was represented by only one school, the higher baseline victimization level may reflect pre-existing differences between the two school contexts rather than differences attributable to the intervention. Consequently, the observed reduction in victimization should not be interpreted solely as an intervention effect. These findings should be considered preliminary and interpreted cautiously, particularly given the potential contribution of baseline imbalance, regression to the mean, and unmeasured school-level differences.

The findings are particularly relevant within the Turkish educational context. Recent national evidence from the TIMSS 2023 Turkey Report indicates that exposure to peer bullying among fourth-grade students has increased compared with the previous assessment cycle, while verbal and relational forms of bullying remain the most common victimization experiences [10]. These findings reinforce the importance of implementing evidence-based preventive programs during the primary school years, before maladaptive peer interaction patterns become established. The present study addresses this need by providing one of the first pilot school-level randomized evaluations of a culturally adapted, game- and drama-based bullying-prevention program developed for Turkish primary school children.

The process evaluation provided preliminary evidence regarding the feasibility of delivering the Renkli Adımlar program in the participating school. All six planned sessions were delivered as scheduled, no major deviations from the intervention protocol were documented, no intervention-related adverse events were reported, and all enrolled participants completed the post-intervention assessment. Teacher feedback also indicated positive perceptions regarding student engagement, the appropriateness of the activities, and the implementation process. However, these findings should be interpreted cautiously as indicators of feasibility rather than as evidence of high acceptability. Attendance was monitored and participants included in the intervention analysis were reported to attend sessions regularly, but detailed session-level attendance or adherence rates were not quantified in the present report. In addition, acceptability was assessed descriptively through feedback from only two teachers, without a formal student acceptability measure or structured quantitative acceptability scale. Therefore, the present findings support the feasibility of delivering the intervention under the conditions of this pilot study, while the acceptability of the program remains preliminary and requires more systematic evaluation in future studies using quantitative attendance and adherence indicators, structured student and teacher feedback, and other predefined feasibility measures.

### Strengths and Limitations

This study has several important strengths. First, to our knowledge, it is one of the few pilot school-level randomized studies conducted in Türkiye evaluating a structured game- and drama-based intervention specifically designed to prevent peer bullying and promote empathy among primary school children. Second, the intervention was delivered using a standardized manual, thereby enhancing consistency across sessions and facilitating future replication. Third, the use of interactive educational methods, including creative drama, role-playing, puppet activities, cooperative learning, and classroom discussions, increased the ecological validity of the intervention by reflecting authentic classroom practices. Finally, the study was registered on ClinicalTrials.gov retrospectively after completion of data collection. The study had received ethics approval before implementation, and the primary study variable and an additional outcome related to empathy had been prespecified in the ethics committee research application submitted prior to data collection.

Several limitations should be considered when interpreting the findings. First and most importantly, only two schools were included, with one allocated to the intervention condition and one to the control condition. Because the school and intervention assignments were perfectly aligned, the observed between-group differences may reflect pre-existing or unmeasured school-level characteristics rather than the Renkli Adımlar intervention itself. Although school-level randomization was used to minimize potential contamination between study conditions, randomization of only two schools cannot adequately balance contextual characteristics across conditions or disentangle intervention effects from school-level variation. Therefore, the statistically significant group × time interactions observed for bullying and victimization should be considered preliminary and exploratory rather than definitive evidence of a causal effect of the intervention. Importantly, the principal limitation is not simply the inability to estimate the intracluster correlation coefficient (ICC), but the fundamental inability to separate the intervention effect from the school effect in a two-cluster design. Future cluster-randomized trials should include multiple schools per condition to provide more robust estimates of intervention effects and school-level variation.

Second, the study did not include an attention- or activity-matched control condition. Students in the control school continued their usual school activities, whereas students in the intervention school received six structured face-to-face sessions delivered by the second, third, and fourth authors using interactive educational activities. Consequently, the observed between-group differences may partly reflect nonspecific effects associated with novelty, increased researcher attention, additional classroom interaction, or participation in structured group activities rather than the specific content of the Renkli Adımlar program. Future trials should consider an attention-matched or active-control condition that provides a comparable amount of researcher contact, classroom interaction, and structured activity, without the specific intervention components.

Third, outcomes were assessed immediately after the intervention, precluding evaluation of the long-term sustainability of behavioral changes. Fourth, bullying and empathy were assessed using self-report instruments, which may be subject to recall and social desirability biases. Fifth, participants were recruited from public primary schools within a single province of Türkiye, which may limit the generalizability of the findings to other educational settings and cultural contexts. Finally, although standardized intervention procedures were followed, objective fidelity measures such as independent observations were not collected. Future multicenter cluster-randomized controlled trials should therefore include multiple schools per condition, longer follow-up periods, active comparison conditions, and comprehensive fidelity assessments to establish the effectiveness and sustainability of the Renkli Adımlar program.

Another limitation is that student recruitment took place after schools had been allocated to the intervention or control condition. This sequence may have introduced a risk of selection bias, as awareness of the assigned condition could have influenced students’ or parents’ decisions to participate.

## 5. Conclusions

Overall, the findings provide preliminary evidence regarding the feasibility of implementing the Renkli Adımlar program and suggest potentially favorable patterns in bullying-related outcomes. However, given the pilot design and the inclusion of only two school clusters, these findings should be interpreted as preliminary and hypothesis-generating rather than definitive evidence of the intervention’s effectiveness. Larger cluster-randomized trials involving multiple schools per condition are needed to determine the program’s effectiveness while accounting for school-level variation.

## Figures and Tables

**Figure 1:**
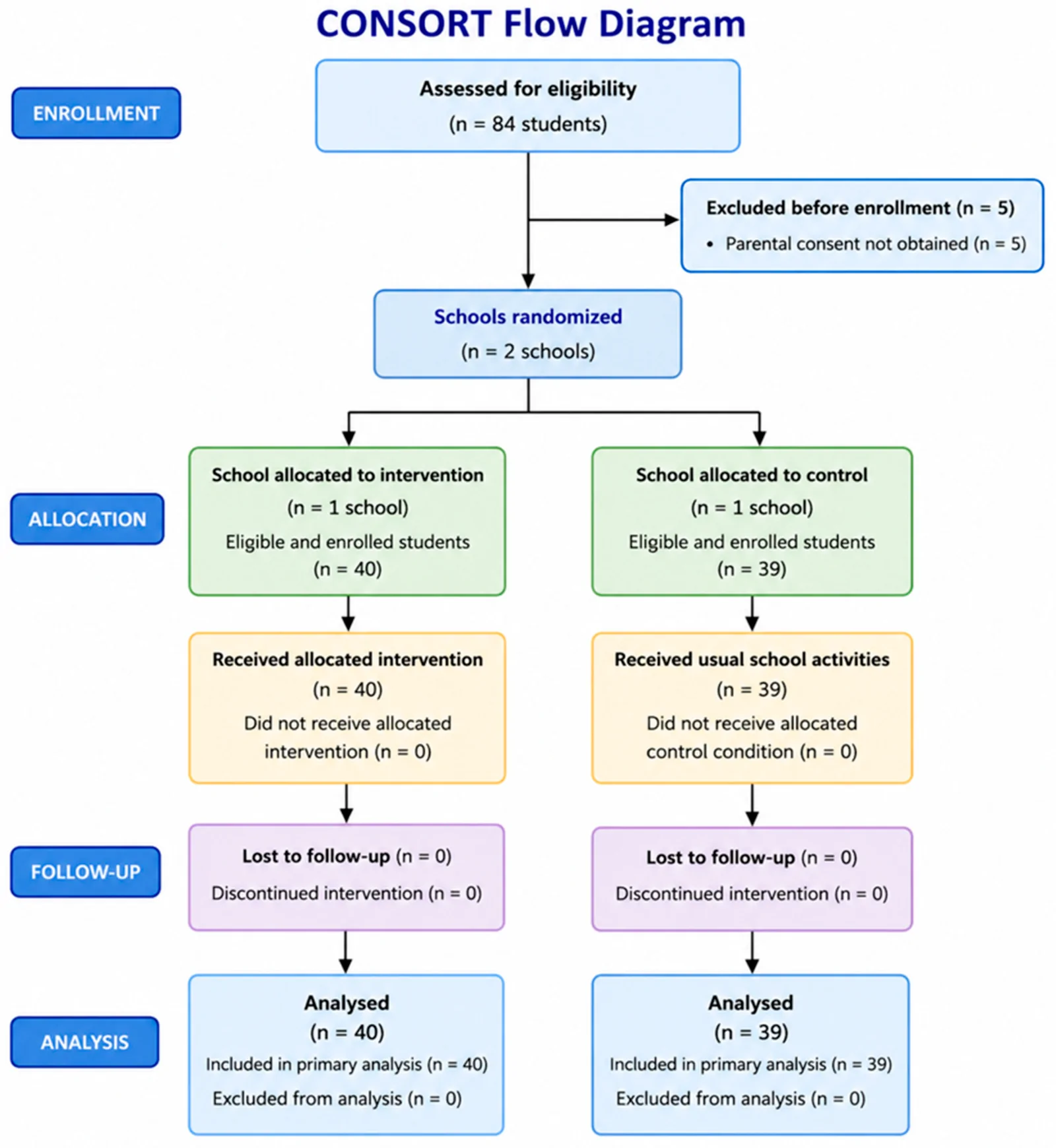
CONSORT flow diagram of participant recruitment, randomization, follow-up, and analysis.

**Figure 2:**
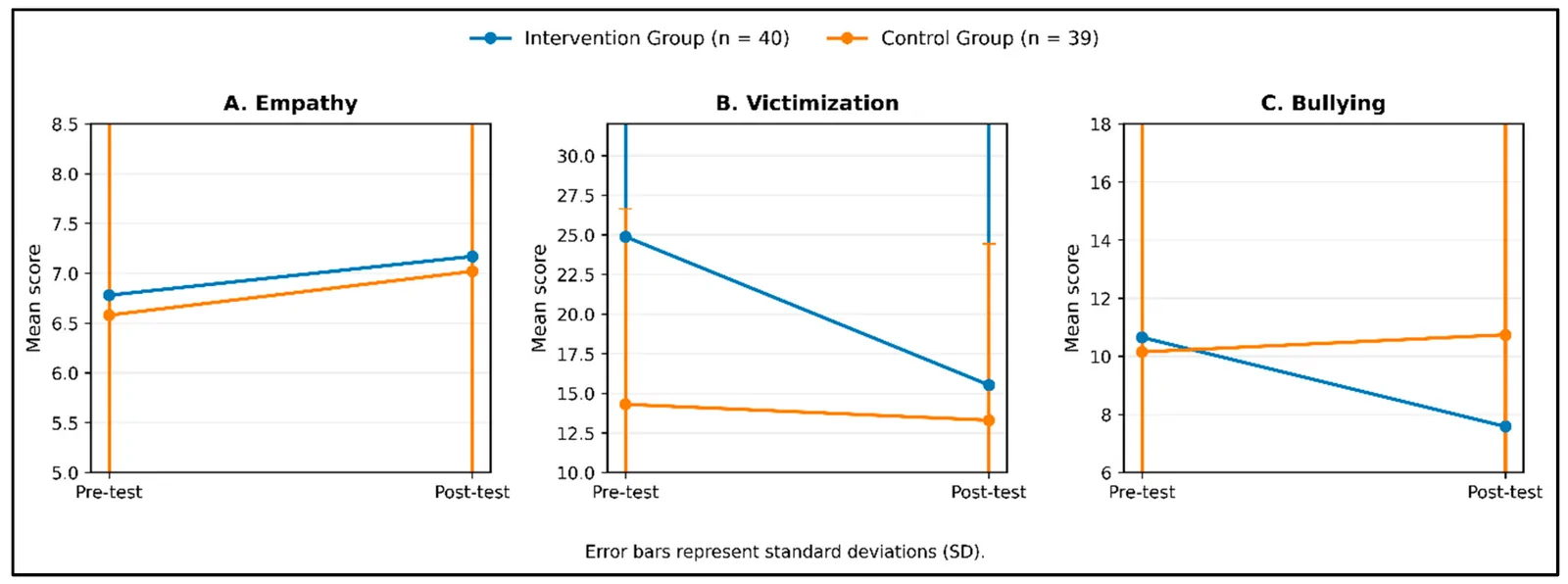
Changes in empathy, victimization, and bullying scores from pre-test to post-test in the intervention and control groups. Values are presented as mean ± SD. Error bars represent standard deviations. Higher empathy scores indicate greater empathy, whereas higher PBIS-CF victimization and bullying scores indicate greater victimization and bullying, respectively.

**Table 1:** Structure and content of the Renkli Adımlar intervention.

Session	Module	Learning Objectives	Teaching Methods	Expected Outcomes
Week 1	Orientation and Baseline Assessment	Introduce the study, establish rapport, explain classroom rules, obtain assent, and complete baseline measures	Program introduction, study orientation, introductory activities, and administration of the IECA and PBIS-CF.	Participant engagement and baseline measurements
Week 2	Bullying Awareness Wheel	Recognize bullying types	Wheel game, discussion	Increased bullying awareness
Week 3	Cartoon and Scenario Analysis	Perspective taking	Animation, discussion	Improved emotional recognition
Week 4	Puppet Role-play	Coping skills	Puppet theatre, role-play	Improved empathy and help-seeking skills
Week 5	Kindness Box	Positive communication	Cooperative learning	Increased prosocial behavior
Week 6	Cyberbullying Awareness	Safe internet use	Scenario discussion	Increased digital citizenship
Week 7	Stop–Think–Act-Cooperative Problem-solving	Strengthening problem-solving, teamwork, and conflict resolution skills	Drama, role-play, cooperative puzzle activity	Improved conflict resolution and cooperation
Week 8	Post-intervention Assessment and Closure	Review the program, reinforce key messages, collect post-test data, and conclude the intervention	Group reflection, feedback, IECA, PBIS-CF	Intervention evaluation and program closure

**Table 2:** Baseline characteristics of participants according to study group.

		IG		CG		Statistics	
		*n*	%	*n*	%	Test Value	*p*-Value
Sex	Girl	24	60	21	53.8	χ^2^ = 0.30	0.58
	Boy	16	40	18	46.2		
Maternal education	Primary school	16	40	10	25.6	χ^2^ = 2.29	0.31
	High school	17	42.5	18	46.2		
	University	7	17.5	11	28.2		
Paternal education	Primary school	13	32.5	9	23.1	χ^2^ = 1.32	0.51
	High school	15	37.5	14	35.9		
	University	12	30	16	41		
Number of siblings	0	3	7.5	6	15.4	χ^2^ = 2.45	0.65
	1	16	40	17	43.6		
	2	14	35	12	30.8		
	3	5	12.5	2	5.1		
	≥4	2	5	2	5.1		
		mean	sd	mean	sd	test value	*p*-Value
Age (years)		9.37	0.58	9.46	0.60	Z = −0.65	0.51
PBIS-CF bullying score		10.65	14.02	10.15	16.36	Z = −0.97	0.37
PBIS-CF victimization score		24.87	24.55	14.30	12.34	Z = −1.76	0.07
IECA score		6.78	2.06	6.58	2.18	Z = −0.62	0.52

χ^2^ = chi-square test, Z = Mann–Whitney U test, sd = standard deviation, IG = intervention group, CG = control group, Index of Empathy for Children and Adolescents (IECA), Peer Bullying Identification Scale–Child Form (PBIS-CF).

**Table 3:** Changes in empathy, victimization, and bullying scores according to mixed repeated-measures ANOVA.

Outcome	Group	Pre-Test Mean ± SD	Post-Test Mean ± SD	Mean Change	Between-Group Difference in Change (95% CI)	F(df)	*p*	η^2^p
PBIS-CF-B	IG	10.65 ± 14.02	7.58 ± 10.57	−3.08	−3.66 (−7.32 to −0.01)	3.959 (1, 77)	0.050	0.049
	CG	10.15 ± 16.36	10.74 ± 16.58	0.59				
PBIS-CF-V	IG	24.87 ± 24.56	15.52 ± 16.53	−9.35	−8.35 (−16.80 to −0.10)	3.870 (1, 77)	0.040	0.048
	CG	14.30 ± 12.34	13.30 ± 11.12	−1.00				
IECA	IG	6.78 ± 2.06	7.17 ± 2.71	+0.39	−0.05 (−0.16 to 0.06)	0.882 (1, 77)	0.351	0.011
	CG	6.58 ± 2.18	7.02 ± 2.21	+0.44				

**Abbreviations:** IG = Intervention group; CG = Control group; SD = Standard deviation; IECA = *Index of Empathy for Children and Adolescents*; PBIS-CF-B = *Peer Bullying Identification Scale–Child Form, Bullying subscale*; PBIS-CF-V = *Peer Bullying Identification Scale–Child Form, Victimization subscale*. The prespecified primary outcome was peer bullying, assessed using the PBIS-CF, with bullying perpetration and victimization examined as separate subscales. Empathy was prespecified as a secondary outcome. The group × time interaction constituted the primary analysis for these outcomes, whereas empathy was prespecified as a secondary outcome. Wilcoxon signed-rank tests were performed as supportive exploratory within-group analyses. Mean change was calculated as post-test minus pre-test; negative values indicate reductions in bullying and victimization scores. Between-group differences were calculated as intervention group minus control group.

**Table 4:** Within-Group Changes in Outcome Scores Based on Wilcoxon Signed-Rank Tests.

Outcome	Group	Z	*p*	r
PBIS-CF-B	IG	−2.13	0.033	0.34
	CG	−1.29	0.199	0.20
PBIS-CF-V	IG	−2.80	0.004	0.44
	CG	−0.54	0.588	0.09
IECA	IG	−2.38	0.017	0.38
	CG	−1.45	0.143	0.23

Z = Wilcoxon signed-rank test statistic; r = effect size. The Wilcoxon signed-rank test was used to examine within-group pre-test-to-post-test changes. These analyses were considered secondary/supportive and exploratory and do not replace the primary group × time comparisons from the mixed repeated-measures ANOVA.

## Data Availability

The datasets generated and analyzed during the current study are available from the corresponding author on reasonable request.

## Supplementary Materials

The following supporting information can be downloaded at: https://www.mdpi.com/article/10.3390/healthcare14193182/s1, Figure S1. Representative educational materials and classroom activities used in the Renkli Adımlar intervention.
