# Supplementary material for: Feasibility and Preliminary Effects of the Renkli Adımlar Game- and Drama-Based Program for Preventing Peer Bullying and Promoting Empathy: A Pilot School-Level Cluster Randomized Controlled Trial
Source: Healthcare (Basel). 2026 Sep 25;14(19):3182. doi: 10.3390/healthcare14193182 (PMC13665273; doi:10.3390/healthcare14193182)
Supplement: Supplementary file 1 [file healthcare-14-03182-s001.zip › healthcare-4480457-supplementary.pdf]

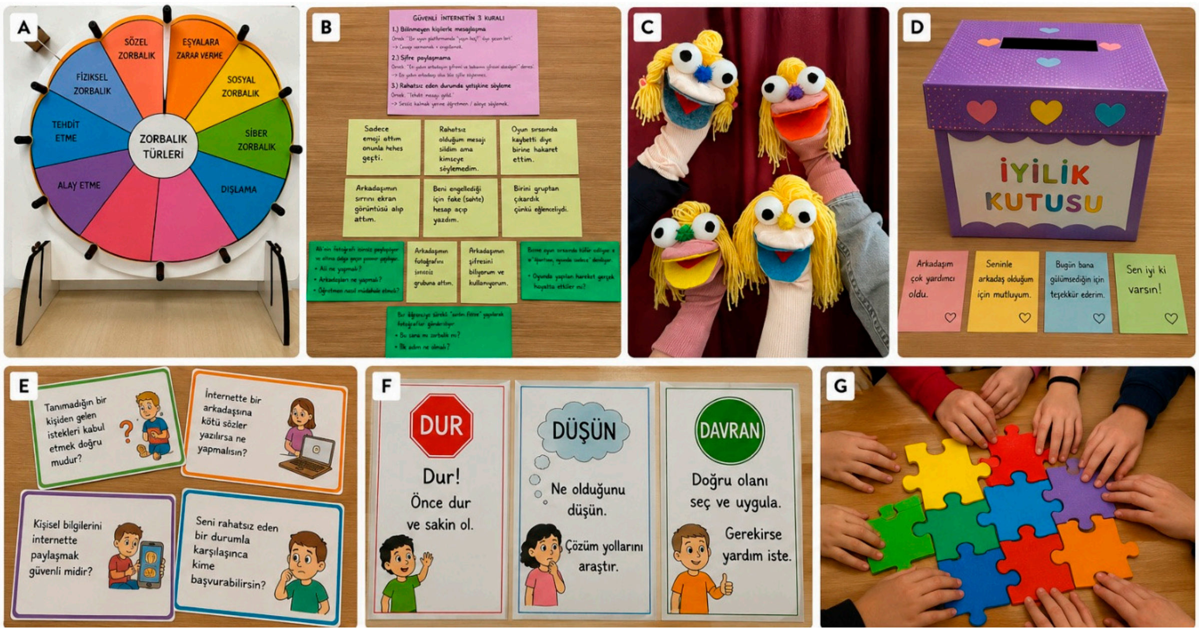

Supplementary Figure S1. Representative educational materials and classroom activities used in the Renkli Adımlar intervention.

(A) Bullying Awareness Wheel used to introduce different forms of bullying. **(B)** Cartoon/animation analysis activity used to facilitate emotional recognition and perspective-taking. **(C)** Puppet role-play activity designed to practice coping strategies and help-seeking behaviors. **(D)** Kindness Box activity promotes positive communication and prosocial behavior. **(E)** Cyberbullying and safe communication activity focusing on responsible digital citizenship. **(F)** Stop–Think–Act (Dur–Düşün–Davran) visual model used to teach structured problem-solving. **(G)** Cooperative group activity reinforces teamwork and empathy through collaborative problem-solving
